# SARS Transmission, Risk Factors, and Prevention in Hong Kong

**DOI:** 10.3201/eid1004.030628

**Published:** 2004-04

**Authors:** Joseph T.F. Lau, Hiyi Tsui, Mason Lau, Xilin Yang

**Affiliations:** * Chinese University of Hong Kong, Hong Kong, China

**Keywords:** SARS, source, transmission, Hong Kong, Chinese

## Abstract

We analyzed information obtained from 1,192 patients with probable severe acute respiratory syndrome (SARS) reported in Hong Kong. Among them, 26.6% were hospital workers, 16.1% were household members of SARS patients and had probable secondary infections, 14.3% were Amoy Garden residents, 4.9% were inpatients, and 20.1% were contacts of SARS patients who were not family members. The remaining 347 case-patients (29.1%) did not have “known” sources of infection. Excluding those <16 years of age, 330 patients with cases from “undefined” sources were used in a 1:2 matched case-control study. Multivariate analysis of this case-control study showed that having visited mainland China, hospitals, or the Amoy Gardens were risk factors (odds ratio [OR] 1.95 to 7.63). In addition, frequent mask use in public venues, frequent hand washing, and disinfecting the living quarters were significant protective factors (OR 0.36 to 0.58). In Hong Kong, therefore, community-acquired infection did not make up most transmissions, and public health measures have contributed substantially to the control of the SARS epidemic.

As of June 11, 2003, a total of 1,755 probable SARS cases were reported in Hong Kong (*1*). Some of the sources of SARS transmission are unknown. For instance, the first major SARS outbreak occurred in the Prince of Wales Hospital in March 2003, and 138 probable cases were reported from March 11 to March 25, 2003 (*2*). Another major outbreak occurred in the Amoy Gardens housing estate on approximately March 26, 2003, and a total of 321 residents were affected (*3*). A total of 381 hospital workers were affected as of May 29, 2003 (*4*). Other sources of infection are possible. Some inpatients were cross-infected by SARS case-patients, who were hospitalized for reasons other than SARS; others may have contracted the disease through known contacts with other SARS patients. The rest of the community-acquired case-patients contracted the diseases through less defined sources. The distributions of the “known” and “undefined” sources of infection have not been reported. Such an initiative would help assess the infectivity and modes of transmission of the virus in the community setting.

Also, reports that public health measures, such as wearing masks, frequent hand washing, avoidance of crowded places, disinfection of the living quarters had been practiced by most of the Hong Kong population during the SARS outbreak (>90%) (*5*). The efficacy of widespread use of masks was controversial (*6*), and evaluating the efficacy of such measures in controlling the epidemic is important.

Our study had two objectives. First, we sought to delineate the distribution of different sources of transmission of the SARS cases in Hong Kong. The number of cases with known and undefined sources was determined. Patients with known sources included those who were hospital workers, those who lived in the Amoy Gardens Estate, those who were probable secondary cases within a household (i.e., those with another household member who had SARS with an earlier date of onset), those who were inpatients and were cross-infected by other inpatients, and those persons who had contact with another SARS patient (who was not one of their household members) before the onset of fever. For the remaining cases, the virus was contracted through undefined sources.

The second objective was to identify the undefined source group. A number of hypotheses were tested to identify relevant risk and protective factors associated with contracting the disease. Risk factors were related to visiting places of potentially high risk and meeting at-risk people. Preventive factors were related to public health measures for prevention.

## Methods

The study population comprised all probable SARS patients whose cases were reported to the Department of Health on or before May 16, 2003 (n = 1,690). The SARS case definition criteria for SARS cases, used by Hong Kong Hospital Authority, is as follows: radiographic evidence of infiltrates consistent with pneumonia and current temperature >38°C or a history of such at any time in the preceding 2 days, and at least two of the following: history of chills in the past 2 days, new or increased cough, breathing difficulty, general malaise or myalgia, typical signs of consolidation, and known exposure. These criteria for cases are equivalent to those in the World Health Organization’s case definition for probable SARS cases (*7*).

## Data Collection

Telephone numbers, as well as some demographic and clinical background information, for all probable SARS case-patients in Hong Kong (identified on or before May 16, 2003 [n = 1,690]) were obtained from the Department of Health. A team of trained interviewers called all these numbers, briefed the person answering the phone about the nature of the study, and invited their household to join the study. Informed consent was obtained directly from the respondents. The number of SARS patients in the household was ascertained, and the interviewer identified the index patient, the person who had the earliest date of onset of fever if the household had more than one SARS patient. The rest of the SARS patients, those with later onset of illness, were considered as having probable secondary or tertiary cases. When a household had had two or more SARS patients with the same fever onset date (11 households), both were treated as index patients rather than as having probable secondary cases. The information obtained was cross-checked with that obtained from the SARS registry. Ethics approval was obtained from the Ethics Committee of the Chinese University of Hong Kong.

The study was conducted from April 4, 2003, through June 10, 2003. Of the 1,690 probable SARS case-patients reported in Hong Kong as of May 16, a total of 1,214 (72%) SARS case-patients from 996 households were covered by our study. Of the remaining 476 case-patients not covered by this study, 140 case-patients (8.2%) did not have a correct telephone number, 163 (9.6%) could not be contacted after at least five attempts, 163 (9.6%) declined to participate, and 10 (0.6%) were either not in Hong Kong or could not communicate in Chinese or English.

## Study Design

The study is part of a project that also includes an investigation of the secondary attack rate of household members. For the first part of this study, the index case-patients were asked whether they were hospital workers, inpatients before contracting SARS, or residents of the Amoy Gardens. The other respondents were asked whether onset of fever occurred within 10 days of contact with a SARS patient. These four types of SARS cases were classified into the known sources group. The rest of the index case-patients were classified into the undefined source group. In the second part of the study, a 1:2 matched case-control study was conducted for the undefined source group to identify risk and preventive factors associated with SARS transmission in the community setting.

Adults >16 years of age were included in the case-control study (17 case-patients were removed from the analysis). Potential geographically related risk factors studied included whether the case-patient had visited (but not lived in) Amoy Gardens, Prince of Wales Hospital, other hospitals or clinics, or crowded places within 10 days before onset of fever. Other risk factors were related to contact with other groups of people during the same reference period, including medical personnel, hospital visitors, and persons with influenzalike symptoms (who were not SARS case-patients). A number of protective factors were related to relevant public health measures, including the frequency of using a face mask in public venues, the frequency of washing hands each day, and disinfection of living quarters thoroughly during the same period. The same questions were asked to the control group, which was recruited by a random telephone survey. Members of the control group were matched for age and sex with the case-patient.

The reference period was the same as that of the matched case-patient. Random telephone numbers were selected from up-to-date local telephone directories. Interviews were conducted in the evening to avoid overrepresenting those who were not working during the daytime. At least three calls were made before an unanswered call was considered as a noncontact. Informed consent was obtained before the interviews were conducted. Almost all case-patients were interviewed within 14 to 28 days after their onset of fever, and the control group was interviewed accordingly. When a participant was unable to answer the questionnaire, a proxy, who was most familiar with the family situation, was interviewed.

## Data Analyses

For the case-control study, odds ratios (OR) were first examined by using univariate logistic regression models. The significant univariate variables were then entered as input for the multivariate forward conditional logistic regression analysis; p values <0.05 were statistically significant. SPSS for Windows Release 11.0.1 (SPSS Inc., Chicago, IL) was used to analyze the data.

## Results

### Cases with Known Sources of Transmission

Of the 1,214 probable SARS cases covered by this study, 22 questionnaires (1.8%) were incomplete and did not allow us to classify the respondents into groups according to source of transmission. The rest (n = 1,192) were analyzed. A total of 192 (16.1%) had probable cases of secondary or tertiary household transmission (Table 1) (i.e., another household member had SARS but fever onset occurred earlier). All the names were verified as being reported to the SARS registry. Another 317 of 1,192 (26.6%) cases were hospital workers; 170 (14.3%) lived in the Amoy Gardens; 58 (4.9%) were inpatients who had been hospitalized for diseases other than SARS and kept on wards with SARS patients. Most infected inpatients were long-term chronic patients and had been hospitalized for >2 weeks before having SARS symptoms. These patients were likely to have been cross-infected. A total of 727 case-patients belonged in one of the four categories (61% of 1,192 cases). Another 240 (20.1%) had come into contact with a SARS patient within a 10-day period before onset of fever. For 347 (29.1%) participants, the source was undefined; these participants were included in the case-control analysis. After excluding 17 case-patients <16 years of age, 330 participants were included in the case-control study.

**Table 1:** Distribution of 1,214 severe acute respiratory syndrome cases covered by the study

	n	%
Incomplete information	22	-
Complete information	1,192	100^a^
Known sources (any of 1.1 to 1.4)	727	61.0
Probable secondary/tertiary household infection	192	16.1
Hospital care workers	317	26.6
Amoy Gardens residents	170	14.3
Inpatients	58	4.9
Not belong to 1.1-1.4 but had contacted SARS patient(s) within 14 days before onset of fever	240	20.1
Patients with cases of undefined sources (do not belong to 1 and 2)	347	29.1
Visited Amoy Gardens^b^	51	4.3
Visited PWH^b^	12	1.0
Visited other hospitals or clinics^b^	134	11.2
Visited an affected country^b^	43	3.6
None of 3.1 to 3.4^b^	118	9.9

^a^Calculated based on complete data. ^b^Not including patients <16 years of age; PWH, Prince of Wales Hospital.

### Univariate Case-Control Analysis

Of the 330 patients with an undefined source of infection, 48% were men and 52% were women. The mean age of the patient group was 47.1 years for both the male and female case-patients (standard deviation [SD] 18.8 and 19.9, respectively, p > 0.05, *t* test). The percentage of participants in the undefined source group in the three periods of the epidemic (before March 25, 2003, from March 26 through April 10, and after April 10) were 24.2%, 36.1%, and 43.5%, respectively.

Members of the patient group were more likely than the control group to have visited mainland China (12.7% vs. 6.5%, p < 0.005). One patient had visited Taiwan, another patient had visited Singapore, two controls had visited Taiwan, and none of the controls had visited Singapore (Singapore and Taiwan were listed as affected areas during the study period). Similarly, patients were also more likely than controls to have visited the Amoy Gardens (15% vs. 2%, OR = 9.10, p < 0.005) (keeping in mind that those who lived in the Amoy Gardens had already been removed from the analysis); patients were more likely than the controls to have visited the Prince of Wales Hospital (3.6% vs. 0.5%, OR = 8.27, p < 0.005) or other hospitals or clinics (40.7% vs. 17.0%, OR = 3.36, p < 0.005) (Table 2). A total of 212 cases of the undefined source group had visited at least one of the above-mentioned categories of places. Frequency of visiting crowded places was, however, not significant in the univariate analysis (21.91% vs. 20.8%, OR = 1.07, p > 0.05).

**Table 2:** Preventive measures and risk factors reported by cases and controls^a^

Factors	Case^b^	Control^c^	Matched univariate OR (95% CI)	Matched multivariate OR (95% CI)	p value^d^	
% visited mainland China (reference=no)	12.7	6.5	2.09 (1.33 to 3.27)^e^	1.95 (1.11 to 3.42)	0.020	
% visited PWH (reference=no)	3.6	0.5	8.27 (2.32 to 29.49)^e^	7.07 (1.62 to 30.75)	0.009	
% visited other hospitals/clinics (reference=no)	40.7	17.0	3.36 (2.49 to 4.54)	3.70 (2.54 to 5.39)	<0.001	
% visited Amoy Gardens (reference=no)	15.5	2.0	9.10 (4.87 to 17.00)^e^	7.63 (3.77 to 15.43)	<0.001	
% visited crowded places frequently (reference=occasionally/seldom/no)	21.9	20.8	1.07 (0.76 to 1.50) NS	-	-	
% contacted someone with fever or influenza (reference=no)	9.0	6.4	1.42 (0.87 to 2.32) NS	-	-	
% social contact with someone who visited a patient in a hospital (reference=no)	8.2	5.2	1.66 (0.96 to 2.85) NS	-	-	
% social contact with medical personnel (reference=no)	7.6	8.6	0.87 (0.52 to 1.44) NS	-	-	
% had a SARS case in the housing estate (reference=no)	6.6	8.5	0.76 (0.44 to 1.31) NS	-	-	
% disinfected the living quarters thoroughly (reference=no)	46.6	74.5	0.30 (0.23 to 0.39)^e^	0.41 (0.29 to 0.58)	<0.001	
Wore a mask in public places frequently (reference=occasionally /seldom/no)	27.9	58.7	0.27 (0.20 to 0.37)^e^	0.36 (0.25 to 0.52)	<0.001	
Washed hands 11 or more times per day (reference=1–10 times/day)	18.4	33.7	0.44 (0.31 to 0.63)^e^	0.58 (0.38 to 0.87)	0.008	

^a^N.S., not significant; OR, odds ratio; CI, confidence interval; -, not used by the multivariate analyses. The reference time period was the 10 days before the date of the patient’s onset of fever. ^b^n = 330. ^c^n = 660. ^d^p values for multivariate OR. ^e^p<0.005.

Members of the case-patient and control groups were not statistically different in the percentage of having come into contact with someone with influenzalike symptoms (those having made contacts with SARS patients were already removed, 9.0% vs. 6.4%, OR = 1.42, p > 0.05). The two groups were also not different in the likelihood of having social contact with someone who had visited a hospital (8.2% vs. 5.2%, OR = 1.66, p > 0.05) or having social contact with medical personnel (7.6% vs. 8.6%, OR = 0.87, p > 0.05). Also patients were not more likely to have a known SARS patient living in the same housing estate, after Amoy Gardens patients had already been removed from the analysis (such data were made available to the public by the government after April 12, 2003) (*8*).

Furthermore, matching for the reference period, members of the case group were less likely than members of the control group to have frequently worn a face mask in public venues (27.9% vs. 58.7%, OR = 0.36, p < 0.005), to have been washed their hands >10 times a day (18.4% vs. 33.7% OR = 0.44, p < 0.005), and to have disinfected their living quarters thoroughly (46.6% vs. 74.5%, OR = 0.30, p < 0.005).

### Multivariate Analysis

When all the variables that were significant in the univariate analysis were used as input for the multivariate stepwise conditional logistic regression analysis, the results show that among the 330 patients with undefined sources, the following were significant risk factors: having visited mainland China (OR = 1.95, p = 0.020, Table 2), having visited the Amoy Gardens (OR = 7.63, p < 0.001), having visited the Prince of Wales Hospital (OR = 7.07, p = 0.009), and having visited other hospitals or clinics (OR = 3.70, p < 0.001) during the reference period. On the other hand, using a mask frequently in public places (OR = 0.27, p < 0.001), washing one’s hands >10 times a day (OR = 0.58, p = 0.008), and disinfecting the living quarters thoroughly (OR = 0.41, p < 0.001) during the reference period were significant protective factors (Table 2).

### Undefined Cases

After removing those case-patients who may have contracted SARS after visiting the Amoy Gardens, the Prince of Wales Hospital, other hospitals, or an affected country, including mainland China, Singapore, and Taiwan (212 cases of the 330 cases), 118 cases remained undefined. They were likely to be community-acquired cases of unknown sources of transmission. When univariate and multivariate conditional logistic regression analyses were repeated for the 118 cases with undefined sources (after 212 patients who had visited some particular places that were associated with risk for transmission were removed from the analysis) and their controls (n = 226), similar results were obtained. The three public health variables—frequently wearing a mask in public places (adjusted OR = 0.36, p < 0.001), washing hands >10 times a day (adjusted OR = 0.44, p = 0.008), and disinfecting the living quarters thoroughly (adjusted OR = 0.36, p < 0.001)—remained significant protective factors. Again, similar to the results of the previous analysis applied to the 330 cases, the other five variables (visiting crowded places, having contact with someone with influenzalike symptoms, having social contact with hospital visitors, having social contact with medical workers, and living with in the same housing estate as other SARS case-patients) were not significant risk factors.

## Discussion

Of the 1,192 participants in this study, approximately 16.1% had probable secondary or tertiary transmission occurring within the household, 26.6% were hospital workers with nosocomial infections, 14.3% were Amoy Gardens patients, and 4.9% were cross-infected inpatients. In 20.1%, SARS might have been contracted when the participant came in contact with a SARS patient who was a nonhousehold member, which may have occurred in a hospital or community setting. SARS may have developed in 17.8% after they visited Amoy Gardens, hospitals or clinics, or affected countries. This computation leaves 9.9% as community-acquired cases of an unknown source.

The percentage of patients related to Amoy Gardens (someone who lived there or visited there) is 18.5% (221/1,192). The percentage of patients with a hospital connection (hospital workers, inpatients, and visitors) is 44.5% (530/1,192). The proportion of unknown community-acquired SARS infection among all SARS cases in this study was considerably lower than the proportion of nosocomial infection, which suggests that preventing hospital outbreaks is essential.

Of the 330 undefined transmissions, 44.2% of the transmissions occurred through hospital visitors. Another study on household transmission also indicated that hospital visits were a significant risk factor for predicting household secondary infection (*9*). Therefore, the severity of future outbreaks, if any, would depend on the ability of the hospital system to control hospital cross-infection and infection of visitors.

Visits to mainland China were associated with SARS transmission, even after adjusting for other variables. Cross-border transmission played a role in the epidemic; although the absolute percentage is not high among the 1,192 case-patients (3.4% or 41/1,192), it is substantially larger among the undefined source group (13.03%). With a case-control design, we could not establish whether this 13.03% was associated with an inflated risk. Cross-border communication and prevention, such as those set in place (temperature screening and health declaration), need to be enforced strictly and consistently. Almost 70% of the 41 participants who visited mainland China had fever onset on or before April 1 (i.e., the early phase of the epidemic) (*5*). None of them had onset after May 3, which is understandable as visiting mainland China was perceived as a high risk by the general public in the late phase of the epidemic (*5*).

The variables related to social contacts (with medical personnel or hospital visitors, with persons with influenzalike symptoms, and with persons living in a housing estate with a reported SARS patient) were not significant. These findings should be interpreted with caution. On one hand, these case-patients should not be stigmatized. On the other, the results may have been confounded because all SARS cases contracted this way were excluded from the analysis. However, confirming that these variables could not account for transmission of the undefined source cases can be useful.

Evidence does not indicate that frequent visits to crowded places were associated with a higher likelihood of community-acquired infection. This finding may remove panic that arose during the epidemic, and daily life need not change as much as it had. Hong Kong is a densely populated city, and it had a large number of SARS cases. The number of community-acquired cases in less populated cities should be much lower than that of Hong Kong. This finding should be interpreted with care as >90% of the general public wore face masks in public places, and >85% avoided visits to public places during the epidemic in Hong Kong (*5*). Although visiting the Amoy Gardens was a risk factor, Amoy Gardens might be the only place where such a large-scale SARS outbreak was attributable to contamination of the environment.

We now have some empirical evidence to suggest that wearing a face-mask frequently in public places, frequent handwashing, and disinfecting one's living quarter were effective public health measures to reduce the risk for transmission (adjusted OR 0.58 to 0.36). The effectiveness of mask use was controversial (*6*). In another study, the prevalence of these three public health preventive public health measures increased significantly from March 21, 2003, to April 1, 2003, (i.e., wearing masks 11.5%–84.3%; frequent hand washing 61.5%–95.1%; home disinfection 36.4%–80%) (*5*). These practices played an essential role in limiting the spread of the virus in the community in Hong Kong.

That disinfecting the living quarter is a strong protective factor has a particular relevance. The reason behind the significance is not completely clear. During the epidemic, the Hong Kong government released frequent announcements of public interest to promote home disinfection using 1:99 bleach water solutions. Most respondents who disinfected their living quarters were probably following the government’s suggestion. Keeping in mind that probable secondary cases had already been removed from the analysis, such protective effect is not referring to the effects that disinfecting the quarter reduced the chance of secondary infection. Environmental contamination (suspected to be related to the sewage system) was reported in the Amoy Gardens, and similar environmental contamination probably did not occur in other places. Such contamination-related infections might be on a small scale and not been noticed. In such circumstances, home disinfection might reduce the risk for transmission. The finding suggests that, in addition to the droplet theory, the fomites theory could not be dismissed.

Our study has a few limitations as well as strengths. First, approximately 72% of all SARS case-patients were included in the study (excluding patients whose contact numbers were incorrect or not available; approximately 78% of those with a valid contact telephone number were included, and the refusal rate was about 10%). The sample size was reasonably large. Second, data were collected retrospectively. Most of the data were, however, collected from the participants within 1 month after onset of fever. Since contracting the disease is a major life event for the patient and family, they should be able to recall whether such factual and benchmark behaviors had been practiced.

The study also has strength of matching for age, sex, and reference time of the behaviors in question, so that both the case and control in a pair were referring to relevant behaviors that occurred within the same 10-day period before the date of onset of fever of the patient. Third, some questions, such as those about disinfection of households or visiting crowed places were nonspecific (the questions asked were “Whether your living quarter had been disinfected thoroughly” and “Whether you had visited crowded places”). Different participants might have defined the terms differently. Further, a number of patients were unable to answer the questions, and a household member who was “most familiar with the household situation” was invited to serve as a proxy. The responses obtained from these informants were compared to those obtained from the patients themselves, and no statistical significance was obtained (p = 0.199 to 0.854) to all variables, except the variable about visiting the Amoy Gardens (p < 0.05).

One particular strength of the study in its evaluation of the three public health measures is that transmissions due to various known sources of infection had been removed as much as possible. In conclusion, the study shows that public health measures may have contributed substantially to the control of SARS epidemic in Hong Kong.
